# Preventing and Controlling Emerging and Reemerging Transmissible Diseases in the Homeless

**DOI:** 10.3201/eid1409.082042

**Published:** 2008-09

**Authors:** Sékéné Badiaga, Didier Raoult, Philippe Brouqui

**Affiliations:** Assistance Publique – Hôpitaux de Marseille, Marseille, France (S. Badiaga, D. Raoult, P. Brouqui); Université de la Méditerranée, Marseille, France (D. Raoult, P. Brouqui)

**Keywords:** homelessness, transmissible diseases, prevention measures, HIV, hepatitis, tuberculosis, scabies, body louse, Bartonella quintana, Rickettsia prowazeckii, perspective

## Abstract

Homelessness is an increasing public health problem. Because of poor living conditions and limited access to healthcare systems, homeless persons are exposed to many communicable infections. We summarize the intervention measures reported to be efficient for the control and the prevention of common transmissible infections among homeless populations. Evidence suggests that appropriate street- or shelter-based interventions for targeted populations are the most efficient methods. Depending on the populations targeted, these interventions may include education, free condom distribution, syringe and needle prescription programs, chest radiography screening for tuberculosis, directly observed therapy for tuberculosis treatment, improvement of personal clothing and bedding hygiene, and widespread use of ivermectin for scabies and body louse infestation. Systematic vaccination against hepatitis B virus, hepatitis A virus, influenza, *Streptococcus pneumoniae*, and diphtheria is strongly recommended. National public health programs specific to homeless populations are required.

## Abstract 2

Appropriate street- or shelter-based interventions for targeted populations are most effective.

Homelessness is an increasing social and public health problem worldwide. According to the United Nations, “absolute homelessness” describes the conditions of persons without physical shelter. “Relative homelessness” describes the condition of those who have a physical shelter but one that does not meet basic standards of health and safety, such as and access to safe water and sanitation, personal safety, and protection from the elements (*1*). An estimated 100 million persons worldwide experience either absolute or relative homelessness (*2*). Homelessness is associated with numerous behavioral, social, and environmental risks that expose persons to many communicable infections, which may spread among the homeless and lead to outbreaks that can become serious public health concerns (*3*–*8*). Epidemiologic studies of homeless populations have reported the following prevalence rates for infectious diseases: 6.2%–35% for HIV infection (*6*,*9*–*13*), 17%*–*30% for hepatitis B virus (HBV) infection (*9*,*10*), 12%*–*30% for hepatitis C virus (HCV) infection (*9*,*10*), 1.2%–6.8% for active tuberculosis (TB) (*3*,*4*), 3.8%–56% for scabies (*11*,*12*), 7%–22% for body louse infestation (*5*,*11*,*13*,*14*), and 2%–30% for *Bartonella quintana* infection (*5*,*15*), which is the most common louse-borne disease in urban homeless.

The prevalence of these transmissible diseases among the homeless varies greatly according to living conditions. Homeless persons who sleep outdoors in vehicles, abandoned buildings, or other places not intended for human habitation are mainly street youth, female street sex workers, and persons with mental health problems (*1*). These persons are frequently injection drug users (IDUs), and they often engage in risky sexual behavior, which exposes them to both blood-borne and sexually transmitted infections such as HIV, HCV, and HBV (*6*,*9*,*10*). Homeless persons sleeping in shelters are mainly single men, but they also include single women, families with children, and mentally ill persons (*1*). The primary health concerns for this population are the overcrowded living conditions that expose them to airborne infections, especially TB (*7*), and the lack of personal hygiene and clothing changes that expose them to scabies, infestation with body lice, and louse-borne diseases (*5*). Homeless persons using single-room hotels or living with friends and family show a high prevalence of illicit drug use and risky sexual behavior that increases the risk for infections transmitted by blood and/or sex (*6*), and they also frequently live in overcrowded conditions that expose them to TB (*7*).

Homeless people face many barriers to accessing healthcare systems; these factors contribute to increasing the spread of infections (*1*). Implementing efficient strategies to survey and prevent the spread of communicable infections among the homeless is a public health priority. Strategies reported to be efficient for controlling or preventing communicable infections in the homeless are targeted interventions that focus on areas where homeless people are more likely to reside and are conducted with a mobile team that includes outreach workers (*8*,*16*–*19*). In this review, which concentrates on the primary communicable infections commonly associated with homelessness, we summarize the main intervention measures reported to be efficient in controlling and preventing these infections.

## Interventions for Homeless at Risk for HIV and Hepatitis

The risk for HIV infection is higher in the following populations of homeless people: those engaged in sexual behavior such as sex work, receptive anal sex, and having multiple sexual partners; those who find it more difficult to use or obtain condoms (*6*,*10*); and those who use drugs in shooting galleries or who share syringes or other drug paraphernalia (*6*). Controlling the spread of HIV among the homeless requires interventions targeting high-risk groups such as youth, female street sex workers, and IDUs (*16*,*19*,*20*). For example, an intensive intervention program targeting homeless youth achieved a significant reduction of unprotected sex acts over 12 months (p = 0.018) and drug use over 12 months (p = 0.019) among female participants as well as a strong reduction in marijuana use over 12 months (p = 0.082) in male participants (*16*). The program involved training shelter staff and residents in small groups, providing access to health resources, and making condoms available easily and at no cost. An intervention program targeting homeless and crack-using African American women provided them with psychoeducational information and skills training on how to reduce HIV risk and drug use; the program significantly (p = 0.03) reduced the number of unprotected sex acts among participants, compared with control participants, at 6 months after the program was started (*19*). In Rhode Island, a prescription program to deliver syringes to high-risk underserved and diverse populations was conducted within the context of comprehensive drug treatment. The program recruited 327 persons and found that 86% saw a physician for syringe prescription at least 1 time, 46% at least twice, and 32% >3 times; this program demonstrated the feasibility and acceptability of such a program for and by its target population, and it reduced the number of injection drug*–*related risky behavior traits (*20*).

Factors predisposing to infections with HBV and HCV are much the same as those for HIV, with HBV a greater risk with of unprotected sex and HCV a greater risk with injection drug–related behavior (*9*,*10*). Therefore, intervention measures to prevent the spread of HCV among the homeless are the same as those noted above for HIV prevention (*16*,*19*,*20*). As for HBV infection, some evidence suggests that HBV immunizations for the homeless are feasible and effective. A study in New Haven, Connecticut, recruited 212 IDUs at syringe-exchange sites to undergo HBV vaccination. Most (63%) were vaccine eligible, including 23% of homeless persons; of the vaccine-eligible IDUs, 77% completed 2 vaccinations and 66% completed all 3 vaccinations (*21*). Homeless IDUs were more likely than other IDUs to complete the vaccination schedule, probably because the vaccination program and the syringe-exchange sites are areas where the homeless tend to congregate, providing more opportunities for them to access these services. An accelerated HBV vaccination schedule should be the regime of choice for homeless people, especially those with a past history of drug use. This recommendation is supported by a study in the United Kingdom that compared completion rates for a conventional HBV vaccine schedule (immunization at 0, 1, and 6 months) conducted among homeless in 1999 with the rates for an accelerated immunization schedule (immunization at 0, 7, and 21 days) in 2000. The completion rates for the accelerated vaccination regimen were almost 7 times higher than rates for the conventional one (*22*). A hepatitis A (HAV) outbreak has been reported among homeless persons and IDUs in Bristol (UK), possibly transmitted parenterally (*8*). The same city is the site of a successful HAV vaccination program for homeless persons and drug users to control HAV outbreaks and prevent transmission to the wider population (*8*). In June 2000, this program immunized 136 homeless persons and IDUs and 9 members of staff in shelters, hostels, drug services, and drop-in centers. The result was a significant (p<0.001) drop in HAV cases in the Bristol population (including homeless persons) from 90 cases (January–June) to 33 (July–December) (*8*).

### Interventions for Homeless at Risk for TB and Airborne Diseases

TB incidence is higher in homeless populations than in the general population, as reported in San Francisco (270 cases/10^5^ persons/year vs. 39.5 cases/10^5^ persons/year) (*23*). Molecular epidemiology studies, using DNA fingerprinting, demonstrated that most TB cases occurring in the homeless are primary infections (*7*,*23*). The spread of TB among the homeless is related to recent person-to-person transmission, which produces outbreaks with large clusters in which >50% of persons are infected (7). Genotyping also identified homeless shelters as major sites of transmission (*7*,*23*). For example, in Los Angeles, California, from March 1994 through May 1999, 3 homeless shelters were sites of TB transmission for 55 (70%) of 79 homeless persons. Thirty-six of these 55 persons were infected in 1 large shelter in which 595 occupants shared 3 sleeping rooms (*7*). Common individual risk factors for TB among homeless persons include alcohol abuse, poor nutrition, and HIV infection (*4*,*23*). Addiction to injection or inhaled drugs has also been reported as associated with TB in the homeless (*4*), but this association remains debated (*7*,*23*).

Interventions to control the spread of TB among homeless persons require early detection of cases and outbreaks in shelters, screening of those persons with whom the infectious person has had contact, and effective treatment of TB patients. According to a shelter-based screening program that used symptom evaluation, chest radiography, and in some cases sputum culture and tuberculin skin testing (TST), a TB infection rate of 1%–3% has been detected among sheltered homeless populations (*24*,*25*). Implementing mandatory shelter-based screening in several homeless shelters in the United States led to reduction of TB transmission among the homeless, as demonstrated by the reduction of genotype clustering in DNA fingerprinting analyses (*17*) (Table 1).

**Table 1:** Communicable infections associated with homelessness*

Specific infections	Transmission route	Risk factors for infection spreading	References
HIV, hepatitis B, STIs	STIs	Sexual risk behavior traits: homosexuality/bisexuality, multiple sexual partners, crack and/or cocaine use, street sex work	(*6*,*9*,*10*)
HIV, hepatitis C, hepatitis B, hepatitis A	Blood-borne infections	Drug risk behavioral traits: sharing syringe, needle, and rinse water	(*6*,*8*–*10*)
Tuberculosis, influenza, diphtheria, pneumococcal pneumonia	Airborne infections	Overcrowding in shelters, alcohol abuse, drug addiction, malnutrition, HIV	(*3*,*7*,*17*,*23*–*25*)
Scabies, body louse infestation	Skin infections	Overcrowding in shelters, lack of personal hygiene, poor clothing and bedding hygiene.	(*5*,*11*,*13*–*15*,*18*)
*Bartonella quintana* infection, epidemic typhus	Louse-borne infections	High prevalence of body louse infestation	(*5*,*13*–*15*)

*STIs, sexually transmitted infections.

No consensus has been reached regarding the most effective diagnostic tools for screening for TB among the homeless. Logistically, TST is likely to be the simplest method to use because it requires only nurses and outreach workers (*17*). It was used successfully to identify TB infection in several intervention programs (*17*,*24*,*25*). However, TST lacks specificity, especially in areas where *Mycobacterium bovis* BCG vaccination is common. Spot sputum screening is also logistically easy, feasible, and efficient for identifying unsuspected TB cases in persons in shelters, and it can permit rapid detection of patients with smears that are acid-fast positive (*24*,*25*). However, 50% of TB patients’ smears are negative, and the patients may be difficult to locate after culture results are known because the homeless tend to be very mobile. Screening by chest radiography either periodically in all residents or specifically in symptomatic persons (e.g., chronic coughers) is likely to be the most cost-effective approach, as was demonstrated in a jail setting (*26*). This strategy detected 42 cases of TB in 9,877 homeless persons in Los Angeles (*7*), and 2 cases of active pulmonary TB among 221 persons during a 1-night “snapshot” shelter-based survey (see “Snapshot Interventions,” below) in Marseille, France (*27*).

Screening the contacts made by TB-infected homeless persons is more effective when it focuses on possible sites of transmission such as homeless shelters rather than when it investigates contacts of specific persons. By contrast, shelters and housing records are excellent sources of information for location-based investigation of contacts (*7*,*23*). The benefits for screening are clear: a 10% in increase in the number of chronically homeless persons with active TB who access treatment each year produced a 12.5% decline in future TB cases in this population after 10 years compared with the number expected without this intervention (*28*). The conditions for effectively treating TB in homeless persons include directly observed therapy (DOT) throughout the treatment course to ensure patient compliance and free medical care, including extended hospitalization and stays in convalescent-care institutions (*3*,*4*). Housing-based programs involving DOT have higher completion rates than programs in acute-care hospital settings.

In addition to TB, influenza, pneumococcal pneumonia, and diphtheria have been reported in the homeless (*29*,*30*). Although no reports on interventions to prevent these infections in the homeless have been published, it has been suggested that immunization against these diseases should be planned and delivered easily and at no cost to homeless people since this population is at high risk for outbreaks and severe illness (*29*,*30*).

### Interventions for Scabies, Body Louse Infestations, and Louse-borne Diseases

Scabies is transmitted by person-to-person contact or by contaminated fomites (e.g., clothes, bedding). It is more prevalent in the homeless than in the general population (*11*). The reported prevalence of scabies varies from 3.8% in shelter-based investigations (*11*) to 56.5% among hospitalized homeless persons (*12*). Human infestations with body lice occur when clothes are not changed or washed regularly, and close body-to-body contacts in crowded environments increase person-to-person transmission of body lice (*31*). In sheltered homeless populations, prevalence rates of body lice vary from 7% to 22% (*5*,*11*,*13*,*14*). In very poor hygienic conditions, an infection prevalence of 80% (*18*) and a single infected person carrying up to 600 lice have been reported (*14*,*18*). Scabies and body lice infestation generate severe pruritus, which leads to scratching, which may result in bacterial superinfections (*11*). In addition, the body louse is an efficient vector for *Bartonella quintana, Rickettsia prowazekii*, and *Borrelia recurrentis* (*3*,*5*,*15*,*32*). *Acinetobacter baumanii* has also been isolated from lice (*32*).

Controlling scabies, body louse infestation, and their effects on the homeless is a challenge. Classic therapeutic measures for scabies are based on bathing, followed by application over the entire skin of topical scabicides such as permethrin, lindane, benzyl benzoate, and crotamiton (*33*). Treatment with 200 μg/kg ivermectin, 2 doses administered 2 weeks apart, has been reported to be as effective as a single dose of a topical scabicide (*33*). Treatment of all close contacts and housemates is recommended, as well as careful washing of clothing and bedding (*33*). The therapeutic modality recommended for body lice is frequent changing and cleaning of clothing, including underwear and socks, as well as frequent treatment of bedding with insecticides or by boiling the sheets (*3*,*34*). In Marseilles, during a 4-year study of arthropod-borne infections among homeless people, we tried to treat, immediately and systematically, all persons in shelters who had scabies or body lice through complete clothing change, application of insecticide, administration of ivermectin as recommended, and education of shelter staff to change and treat the bedding frequently (*5*). Despite these efforts, no significant decrease in the prevalence of scabies and body louse infestation was observed during the study (*5*). In this population, a reduction in the prevalence of body lice infestation was seen after 3 doses of oral ivermectin were administered at 7-day intervals, but the effect was transient and disappeared by day 45 (*18*). In addition, a randomized, double-blind, placebo-controlled trial was conducted in Marseilles to evaluate the effect of a single dose of oral ivermectin on reducing the ectoparasite-based pruritus in the sheltered homeless population. This study showed that a single dose of oral ivermectin transiently reduces pruritus (S. Badiaga, unpub. data). These observations suggest that multiple repeated treatments of ectoparasite-based pruritus with ivermectin are an efficient and practical complement to classic therapeutic measures like frequent, complete changes of clothing and bedding to reduce scabies and body lice infestation in the homeless.

The most common louse-borne disease reported in the urban homeless is *Bartonella quintana* infection (*5*). *B. quintana* is a pathogen restricted to humans and was first described as the agent of trench fever during World War I (*34*). Emergence of *B. quintana* among the homeless was recognized in the early 1990s by simultaneous reports of *B. quintana* endocarditis in 3 homeless persons in France (*35*) and *B. quintana* bacteremia in 10 homeless persons in Seattle, Washington, USA (*36*). Subsequent epidemiologic studies showed *B. quintana* seroprevalence rates of 2%–11% among nonhospitalized homeless (*5*,*14*,*15*) and 30% in hospitalized homeless persons (*15*). *B. quintana* bacteremia rates of 5.4% in 930 nonhospitalized homeless persons (*5*) and 14% in 71 hospitalized homeless persons (*15*) have been reported from Marseilles. *B. quintana* DNA has been identified in 101 (14.9%) of 678 lice collected from the sheltered homeless population in Marseille (*5*), as well as in lice collected from homeless persons in Japan (*14*).

*B. quintana* causes trench fever, chronic bacteremia that may last up to 78 weeks, endocarditis in alcoholic persons without previous valvulopathy, and bacillary angiomatosis in HIV-infected persons (*34*). Chronic bacteremia may be identified by blood cultures in homeless persons seen in emergency departments, as reported in a study from Marseilles (*15*). The phenomenon of chronic bacteremia suggests that humans are the natural reservoir of *B. quintana*, as demonstrated by identification of the bacterium in erythrocytes from homeless persons with *B. quintana* bacteremia (*37*). In addition to delousing, which is the best way to prevent louse-borne diseases, antimicrobial drug therapy against bacterial agents may be important for eradicating reservoirs and preventing complications such as endocarditis in cases of *B. quintana* infection. A randomized, open, placebo-controlled trial demonstrated significant efficacy of doxycycline (200 mg orally once a day for 28 days) in combination with gentamycin (3 mg/kg intravenously once a day for 14 days) in homeless persons with *B. quintana* chronic bacteremia (*38*). A regimen of gentamycin for 14 days and doxycycline for 45 days is recommended for patients with endocarditis (*34*).

Epidemic typhus caused by *R. prowazekii* and relapsing fever due to *Borrelia recurrentis* are 2 other louse-borne diseases that tend to affect the urban homeless. Outbreaks of epidemic typhus occur when body louse infestations are more prevalent in the population, as observed in Burundi (*31*). To date, no outbreak of epidemic typhus or relapsing fever has been observed in the urban homeless, nor has evidence of *R. prowazekii* or *B. recurrentis* been found in lice collected from this population. Nevertheless, during a 4-year study of louse-borne diseases among sheltered homeless persons in Marseille, we detected a sporadic acute autochthonous case of epidemic typhus in a sheltered homeless person (*39*) and significantly higher seroprevalences of *R. prowazekii* antibodies (0.75% vs. 0% in blood donors, p = 0.05) and of *B. recurrentis* (1.61% vs. 0% of blood donors, p = 0.005) (*5*). In a massive outbreak of epidemic typhus observed in Burundi, doxycycline was efficient in controlling the outbreak among jail inmates, causing a decrease in the death rate from 15% to 0.5% after administration of a single dose of 200 mg (Table 2).

**Table 2:** Interventions to control and prevent the spread of infections in the homeless*

Infections and specific interventions	References
HIV, HCV, HBV infections	
Tailored education of targeted population on reducing infection risk with provision of free condoms	(*16*,*19*)
Syringe prescription program and needle exchange programs	(*20*)
HBV, HAV infections	
HBV accelerated immunization	(*21*,*22*)
HAV immunization	(*8*)
Tuberculosis	
Shelter based-intervention with chest radiograph screening, sputum culture, tuberculin skin testing	(*17*,*24*,*25*,*27*)
Genotyping	(*7*,*23*)
Contact investigation through homeless shelters	(*7*,*23*)
Influenza, diphtheria, *Streptococcus pneumoniae* infections	
Systematic vaccination	
Scabies, body louse infestation	
Providing facilities for bathing and laundry; insecticide application to bedding in shelters	(*5*,*34*,*35*)
Ivermectin for scabies, body louse, and ectoparasite-based pruritus	(*18*,*34*)
Louse-borne diseases	
Doxycycline and gentamicin for persons with chronic *Bartonella quintana* bacteremia	(*39*)
Doxycycline for persons with epidemic typhus	(*32*)

*HCV, hepatitis C virus; HBV, hepatitis B virus; HAV, hepatitis A virus.

## Snapshot Interventions

Yearly snapshot interventions in shelters, performed by large multidisciplinary teams, have been reported to be efficient for controlling or preventing infections among the homeless (*5*,*11*). These investigations can reach a category of homeless who do not usually seek healthcare. In Marseilles, since 2000, a large mobile team is sent once a year to perform these snapshot interventions in order to survey louse-borne disease in the 2 shelters designated for accommodating the homeless (*5*). This team comprises 30–40 persons, including physicians, residents, or fellows; nurses; and outreach workers. During interventions, homeless persons who choose to participate are interviewed and physically examined. Clothes are carefully screened for body lice, and specific treatment is given when appropriate. Nurses also collect blood and other microbiologic samples for serologic tests for louse-, flea-, and tick-borne diseases, as well as hepatitis, HIV; and syphilis. Arthropods are collected and PCR-screened for pathogens such as *Bartonella*, the epidemic typhus rickettsia, and *Borrelia recurrentis*. Depending on the epidemiologic situation, other surveys such as for the prevalence of TB and other respiratory diseases can be organized (*27*). In such cases, a pneumologist and radiography technologists using a mobile x-ray machine are added to the intervention team. These snapshot interventions led to successively identifying a high prevalence of louse infestation, louse-borne diseases such as *B. quintana* infection, and skin infections among this homeless population (*5*,*11*). Snapshot interventions have also identified the risk for the homeless of acquiring other louse-borne diseases such as epidemic typhus and relapsing fever, and enabled the first isolation of *A. baumanii* from lice (*5*,*32*,*39*). This strategy of wide systematic testing of infectious diseases in this population also led to the unexpected discovery of an outbreak of acute Q fever in a homeless shelter in Marseille (*40*). Epidemiologic investigations of this outbreak showed that exposure to wind from an abandoned slaughterhouse, used for an annual Muslim sheep feast, was the main risk factor for developing *Coxiella burnetii* infection (Figure).

**Figure:**
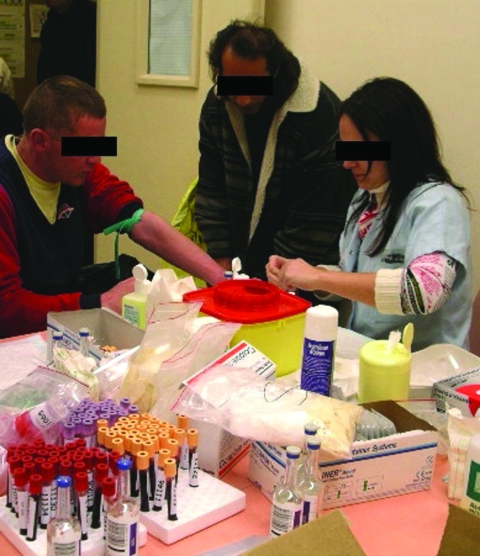
An intervention in a homeless shelter in Marseilles for infectious diseases survey.

## Conclusions

Evidence suggests that appropriate public health interventions can be effective in preventing and controlling the spread of numerous transmitted diseases among homeless persons, which is a public health concern both for the homeless and the larger population. These interventions should be tailored to the targeted populations and focused on areas where the homeless are more likely to reside. The strategies reported to be efficient include tailored education; distribution of free condoms; implementation of a syringe and needles prescription program for HIV and HCV; systematic chest radiography for TB screening in shelters and DOT for TB; improvement of personal, clothing, and bedding hygiene; use of ivermectin to treat pruritus most often caused by scabies or body louse infestation; and immunizations against HBV, HAV, influenza, *Streptococcus pneumoniae,* and diphtheria. Implementation of systematic vaccination schedules to prevent communicable diseases in the homeless is a major public health priority. The success of these interventions requires the implementation of a national public health prevention program for the homeless. A yearly snapshot intervention is 1 means to achieve these objectives.
